# Effectiveness of a cervical pessary for women who did not deliver 48 h after threatened preterm labor (Assessment of perinatal outcome after specific treatment in early labor: Apostel VI trial)

**DOI:** 10.1186/s12884-016-0935-7

**Published:** 2016-07-12

**Authors:** Frederik J. R. Hermans, Ewoud Schuit, Brent C. Opmeer, Martijn A. Oudijk, Mireille Bekker, Mallory Woiski, Caroline J. Bax, Marieke Sueters, Hubertina C. J. Scheepers, Maureen T. M. Franssen, Eva Pajkrt, Ben Willem J. Mol, Marjolein Kok

**Affiliations:** Department of Obstetrics and Gynaecology, Academic Medical Center, Amsterdam, The Netherlands; Julius Center for Health Sciences and Primary Care, University Medical Center Utrecht, Utrecht, Netherlands; Stanford Prevention Research Center, Stanford University, Stanford, CA USA; Clinical Research Unit, Academic Medical Center, Amsterdam, The Netherlands; Department of Obstetrics and Gynaecology, University Medical Center Utrecht, Utrecht, The Netherlands; Department of Obstetrics and Gynaecology, Radboud University Nijmegen Medical Center, Nijmegen, The Netherlands; Department of Obstetrics and Gynaecology, VU University Medical Center, Amsterdam, The Netherlands; Department of Obstetrics and Gynaecology, Leiden University Medical Center, Leiden, The Netherlands; Department of Obstetrics and Gynaecology, Maastricht University Medical Center, Maastricht, The Netherlands; Department of Obstetrics, University Medical Center, University of Groningen, Groningen, The Netherlands; The Robinson Research Institute, School of Paediatrics and Reproductive Health, University of Adelaide, Adelaide, Australia

**Keywords:** Threatened preterm birth, Treatment, Pessary, Cervical length, Fetal fibronectin

## Abstract

**Background:**

Preterm birth is a major cause of neonatal mortality and morbidity. As preventive strategies are largely ineffective, threatened preterm labor is a frequent problem that affects approximately 10 % of pregnancies. In recent years, risk assessment in these women has incorporated cervical length measurement and fetal fibronectin testing, and this has improved the capacity to identify women at increased risk for delivery within 14 days. Despite these improvements, risk for preterm birth continues to be increased in women who did not deliver after an episode of threatened preterm labor, as indicated by a preterm birth rate between 30 to 60 % in this group of women. Currently no effective treatment is available. Studies on maintenance tocolysis and progesterone have shown ambiguous results. The pessary has not been evaluated in women with threatened preterm labor, however studies in asymptomatic women with a short cervix show reduced rates of preterm birth rates as well as perinatal complications.

The APOSTEL VI trial aims to assess the effectiveness of a cervical pessary in women who did not deliver within 48 h after an episode of threatened preterm labor.

**Methods/Design:**

This is a nationwide multicenter open-label randomized clinical trial. Women with a singleton or twin gestation with intact membranes, who were admitted for threatened preterm labor, at a gestational age between 24 and 34 weeks, a cervical length between 15 and 30 mm and a positive fibronectin test or a cervical length below 15 mm, who did not deliver after 48 h will be eligible for inclusion. Women will be allocated to a pessary or no intervention (usual care). Primary outcome is preterm delivery < 37 weeks. Secondary outcomes are amongst others a composite of perinatal morbidity and mortality. Sample size is based on an expected 50 % reduction of preterm birth before 37 weeks (two-sided test, α 0.05 and β 0.2). Two hundred women with a singleton pregnancy need to be randomized. Analysis will be done by intention to treat.

**Discussion:**

The APOSTEL VI trial will provide evidence whether a pessary is effective in preventing preterm birth in women who did not deliver 48 h after admission for threatened preterm labor and who remain at high risk for preterm birth.

**Trial registration:**

Trial is registered at the Dutch Trial Register: http://www.trialregister.nl, NTR4210, date of registration: October 16th 2013.

## Background

Preterm birth, defined as birth before 37 weeks gestational age, affects 5-12 % of pregnancies in the developed world [1, 2]. Since neonatal mortality and severe morbidity rates are inversely proportional with gestational age, preterm birth is held accountable for 75 % of neonatal mortality and 70 % of short and long-term neonatal morbidity [1, 2]. Despite all efforts to reduce the incidence of spontaneous preterm birth, rates remain constant making preterm birth a major challenge for obstetric healthcare professionals.

Threatened preterm labor often precedes preterm birth and is a heterogeneous clinical condition in which labor seems to start before 37 weeks gestational age [3]. It presents itself by frequent uterine contractions leading to cervical changes or by preterm prelabor rupture of the membranes (PPROM). Approximately 10 % of all pregnant women experience an episode of threatened preterm labor requiring hospital admission [4]. Cervical length and fetal fibronectin (fFN) testing are used to differentiate between women at an increased risk for preterm delivery and those who are unlikely to deliver [5, 6]. The APOSTEL-I study showed that women with a cervical length less than 15 mm or a cervical length between 15 and 30 mm and a positive fFN test result are at increased risk for preterm delivery within seven days. In addition, the risk of preterm birth in women who do not deliver within the first seven days continues to be increased (30 to 60 %) in the subsequent weeks of pregnancy [4, 5, 7–9].

Currently, women with threatened preterm labor before 34 weeks gestational age and a high predicted risk for delivery at short-term, usually defined as delivery within seven days, are treated with tocolysis and antenatal corticosteroids to improve neonatal outcome [10]. No effective treatment has been established yet for women who did not deliver, although these women still remain at an increased risk (25 %) to deliver before 34 weeks when cervical length is < 15 mm or cervical length is ≥15 and < 30 mm with a positive fFN test result, respectively [11].

The APOSTEL-II trial showed no beneficiary effect of maintenance tocolysis with nifedipine compared to placebo on a composite of adverse perinatal outcome [12].

Results on progesterone as maintenance tocolysis are contradictive. A Cochrane review found no differences in reduction of preterm birth or neonatal outcomes [13]. Whilst another recently published systematic review, including other studies, did find a significant benefit from vaginal progesterone in reduction of preterm birth and prolongation of pregnancy [14]. However, both systematic reviews did not include the largest study available on vaginal progesterone in women with preterm labor that concluded that there was no beneficiary effect [15].

Currently no RCTs have been published investigating a cervical pessary in women with threatened preterm labor. However, results in asymptomatic women with short cervical length and singleton as well as multiple pregnancies look promising, with reported reduction of poor perinatal outcome for singleton (RR 0.14 (95 % CI; 0.01 to 0.39)) and multiple pregnancies (RR 0.23 (95 % CI; 0.09 to 0.60)) [16, 17]. Additionally, in these women with a short cervical length decreased rates were found for delivery < 34 weeks in singleton pregnancies (RR 0.18 (95 % CI; 0.08 to 0.37)) and delivery < 32 weeks in multiple pregnancies (RR 0.44 (95 % CI 0.20 to 0.98)).

In conclusion, women who do not deliver after an episode of threatened preterm labor remain at increased risk of preterm birth in the subsequent weeks of pregnancy [11] and as of yet there is no effective treatment to prevent a preterm delivery in these women Therefore the objective of the APOSTEL VI trial is to investigate whether treatment with a cervical pessary is effective in reducing preterm birth in women who remain at high risk for preterm birth after an episode of threatened preterm labor, in which they did not deliver.

## Methods/Design

### Aim

The aim of the APOSTEL VI trial is to evaluate whether treatment with a cervical pessary is effective in reducing preterm birth < 37 weeks in women who have not delivered after an episode of threatened preterm labor between 24^+0^ and 34^+6^ weeks gestational age but remain at increased risk for preterm delivery.

### Participants/eligibility criteria

A woman, ≥ 18 years, becomes eligible 48 h after primary admission for threatened preterm labor and subsequent inclusion and randomization must take place within the following 72 h. Within the first 48 h of admission treatment with antenatal corticosteroids and tocolysis will be applied according to the national preterm labor guideline by the Dutch College of Obstetricians and Gynaecologists (NVOG) [10].

Women who did not deliver after a 48-h episode of threatened preterm labor will be eligible for randomization if they comply with the following criteria:

#### Inclusion criteria

- o Threatened preterm labor as defined from;
-  
o Cervical length less than 15 mm (<15 mm)

*OR*

-  
o Cervical length between 15 and 30 mm (≥15 mm and < 30 mm) AND a positive fibronectin test result
- o No delivery within 48 h after admission for threatened preterm labor
- o Singleton or twin gestation
- o Gestational age between 24^0/7^ and 34^6/7^ weeks with intact membranes.

#### Exclusion criteria

- o Ruptured membranes
- o Signs of intra-uterine infection (maternal fever, tachycardia at Cardiotocography (CTG))
- o Signs of fetal distress at CTG
- o Known major fetal anomalies
- o Cervical dilatation ≥3 cm
- o >72 h elapsed after becoming eligible to participate
- o Residual cervical length that makes it impossible to place a pessary

**Fig. 1:**
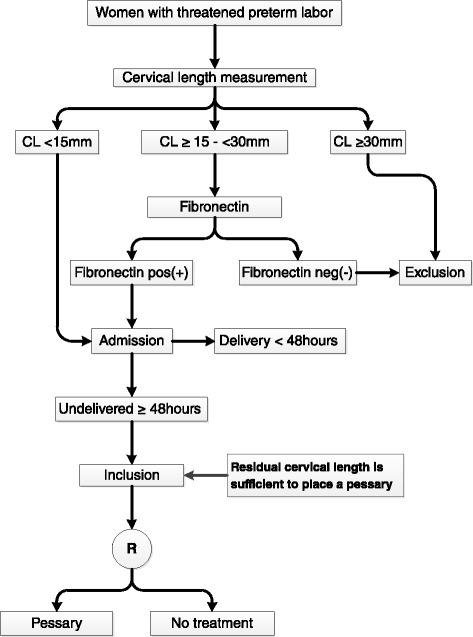
Flowchart Apostel VI

A flow diagram for patient selection can be found in Fig. 1.

### Procedures, recruitment, randomization and collection of data

This is a nationwide multicenter open-label randomized clinical trial in eight perinatal centers with neonatal intensive care unit (NICU) facilities in The Netherlands who collaborate in the Dutch Consortium for Healthcare Evaluation and Research in Obstetrics and Gynecology - NVOG Consortium 2.0 (www.studies-obsgyn.nl). The Dutch Obstetric Consortium is a research collaboration of obstetric clinics in the Netherlands. All centers will be responsible for identification, recruitment and randomization of eligible women.

All women admitted for threatened preterm labor will receive written study information, available in Dutch and English and will be counselled by Good Clinical Practice trained nurses, midwives or doctors. After written informed consent, eligible women will be randomized to treatment with a cervical pessary or no treatment (usual care) through a web based application hosted by the Clinical Trials Unit of the Academic Medical Center, Amsterdam, according to a computer-generated randomization sequence in a 1:1 ratio, with a random block size with a maximum of four. Randomization will be stratified by type of gestation (singleton versus twin pregnancy). Demographical, medical and obstetrical information will be collected at baseline. Baseline information will be merged with relevant information collected during participation in the study in an online case report form (CRF) by GCP trained research nurses and midwives.

### Intervention

Women allocated to a cervical pessary will receive an Arabin® pessary. This is a double ring shaped pessary made of non-allergic, soft and flexible silicone, available in different sizes. During a simple vaginal examination that is not painful, a midwife or gynaecologist will assess which size pessary fits best and subsequently the pessary is folded and placed around the cervix.

The pessary will remain in place until 36 weeks gestational age or until delivery, whatever comes first. Removal of the pessary, reason of removal and reinsertion of a new pessary will be recorded. Apart from pessary placement, all women will receive care according to local protocol.

### Outcome measures

#### Primary outcome measure

The primary outcome will be preterm birth before 37 weeks of gestational age.

#### Secondary outcome measures

Secondary outcomes will be a composite of adverse perinatal outcome, time–to-delivery, gestational age at delivery, preterm birth before 32 and 34 weeks gestational age, birth weight and percentile, days of admission in hospital, NICU admission, maternal morbidity, maternal admission days for preterm labor, re-admittance for new symptoms of preterm labor, side-effects of pessary, days on additional oxygen or supported ventilation and costs. Outcomes will be recorded up until 12 weeks corrected age.

Composite adverse perinatal outcome is defined as perinatal death or severe morbidity. This composite includes: severe respiratory distress syndrome (RDS), bronchopulmonary dysplasia (BPD) according to *Jobe and Bancalari* [18], intraventricular haemorrhage grade III or IV according *de Vries et al.* and *Ment et al.* [19, 20], necrotizing enterocolitis (NEC) > stage 1 according to *Bell et al.* [21], periventricular leucomalacia > grade 1, culture proven sepsis and death before discharge from the hospital.

### Statistical issues

#### Sample size

Based on findings of previous studies we anticipate a reduction of preterm birth < 37 weeks with 50 % [16, 17].. We need to include 180 women in total (two groups of 90 women), with an alpha error of .05 and beta error .2 to show a reduction of preterm birth < 37 weeks from 40 % to 20 %. Assuming a 10 % drop out rate we will need to randomize a total of 200 participants (100 participants per group). Primarily the aim is to include 200 women with a singleton pregnancy. Women with a twin pregnancy will be included as well, however they will not contribute to the number of required inclusions.

#### Data analysis

The analysis will be done by intention to treat. The primary analysis will be limited to singletons only; a secondary analysis will include both women with a singleton and a twin pregnancy. Differences in the main outcome preterm birth < 37 weeks’ between the pessary and no treatment group will be assessed using a random intercept, fixed effects binomial regression model with a log-link function, resulting in a relative risk (RR) with accompanying 95 % confidence interval (CI). In the secondary analysis stratified randomization by type of gestation will be accounted for by adding the type of pregnancy as a covariate to the regression model. In case of equivalence in outcomes between treatment allocations, the analysis will be repeated on a per protocol basis.

Secondary outcomes on the child level in the primary analysis and secondary outcomes on the maternal level in both the primary and secondary analysis will be analyzed equivalent to the primary outcome measure. In the secondary analysis dichotomous outcomes on the infant level will be assessed using a binomial generalized estimating equations model (GEEs) with a log-link function and using an unstructured correlation matrix, resulting in a relative risk (RR) with accompanying 95 % confidence interval (CI). We will account for interdependence between outcomes in twin pregnancies by considering the mother as a cluster variable [22]. Time to delivery will be assessed using Kaplan-Meier analysis with a log-rank test to assess the statistical significance between curves, and a Cox proportional hazards model.

Pre-specified subgroup analysis will be performed on singletons only and is planned based on risk stratification (cervical length < 15 mm versus cervical length ≥15 mm and < 30 mm and positive fetal fibronectin) and obstetric history subdivided on parity and a history of preterm birth (nulliparous versus multiparous with history of preterm birth and multiparous women without a history of preterm birth). Subgroup effects will be investigated for the outcomes preterm birth < 37 weeks and adverse perinatal outcome and will be assessed by including an interaction term between the subgrouping variable and treatment allocation as a covariate to the regression model. When the interaction shows to be statistically significant (*p <* 0.05) a stratified subgroup analysis will be performed to study the effect of treatment in different strata of the subgroup.

#### Interim analysis

An independent Data Safety Monitoring Committee (DSMC) with four members will be formed. Interim analysis will be performed for safety and efficacy after complete data collection of the first 100 women [t = 0.5]. The Peto-stopping rule will be used for testing for efficacy. Given the strong association between gestational age at birth and adverse perinatal outcome there is an opportunity to expand the number of inclusions, if at interim the incidence of the primary outcome turns out to be clinically relevant lower in the pessary group. This is in order to obtain sufficient power to detect a difference in adverse perinatal outcome at the end of the study. Sufficient funds must be available to expand to number of inclusions.

An independent statistician will perform interim analysis and an independent DSMC will advise whether the trial should be stopped or continued.

The data safety monitoring board will be blinded for the treatment allocation, but can be unblinded by opening a sealed envelope. Adverse Events (AEs) and Serious Adverse Events (SAEs) will be reported to the DSMC. If the DSMC feels participating in the trial leads to safety risks, the DSMC can always advice to stop the tria**l.**

## Discussion

Preterm birth is the major cause of neonatal morbidity and mortality. Of all perinatal mortality, 50 % to 70 % is associated with preterm birth. Similarly, neonatal morbidity is inversely proportional to gestational age and thus preterm birth. Since women who do not deliver after an episode of threatened preterm labor remain at an increased risk of preterm birth and currently no effective intervention is available, it is important to evaluate potential treatments for effectiveness. As such, the pessary seems a potential alternative in this group of women.

## Abbreviations

fFN, fetal fibronectin; NICU, neonatal intensive care unit; CRF, case report form; BPD, bronchopulmonary dysplasia; PVL, periventricular leucomalacia; NEC, necrotising enterocolitis; SAE, serious adverse events; SUSAR, suspected unexpected serious adverse reactions; DSMC, data safety and monitoring committee., preterm labor, preterm labor
